# Profile of Users and Adequacy of Hospital Emergency Services in Response to Healthcare Demand Among Population Aged 65 Years and over

**DOI:** 10.3390/diseases13070190

**Published:** 2025-06-21

**Authors:** Rafael Gómez-Galán, José Francisco López-Gil, María Mendoza-Muñoz, Jorge Carlos-Vivas, Julián Carvajal-Gil, Laura Muñoz-Bermejo

**Affiliations:** 1 Research Group on Physical and Health Literacy and Health-Related Quality of Life (PHYQOL), University of Extremadura, 06800 Badajoz, Spain; rgomez@unex.es; 2 School of Medicine, Universidad Espíritu Santo, Samborondón 092301, Ecuador; 3 Vicerrectoría de Investigación y Postgrado, Universidad de Los Lagos, Osorno 5290000, Chile; 4 Department of Communication and Education, Universidad Loyola Andalucía, 41014 Sevilla, Spain; mamendozam@unex.es; 5 Physical Activity for Education, Performance and Health Research Group (PAEPH), Faculty of Sport Sciences, University of Extremadura, 10003 Cáceres, Spain; jorgecv@unex.es; 6 Social Impact and Innovation in Health (InHEALTH) Research Group, Faculty of Sport Sciences, University of Extremadura, 10003 Cáceres, Spain; carvajal@unex.es (J.C.-G.); lauramunoz@unex.es (L.M.-B.)

**Keywords:** emergency health services, age, morbidity, frail elderly individuals, health demand, appropriate resource use

## Abstract

**Objectives**: This study aimed to describe the profile and identify the clinical and sociodemographic factors associated with emergency department (ED) use among patients aged ≥65 years. **Methods**: This was a retrospective cross-sectional study of patients aged ≥65 years who were seen in the emergency department of the Hospital de Mérida (Spain) in 2019, the year before the Coronavirus Disease 2019 (COVID-19) pandemic. Descriptive statistics were calculated for dependent variables such as hours of ED stay, total number of visits, reasons for discharge, and diagnostic specialty, disaggregated by gender, season, age group, day type (work or holiday), shift, and population area (rural or urban). **Results:** Significant differences in ED hours were found according to gender (*p* < 0.001), season (*p* = 0.024), age group (*p* < 0.001), attention shift (*p* < 0.001), and population area (*p* = 0.003). Discharge to home was the most common destination (63.5%), followed by hospital admission (25.7%). Admissions for gastrointestinal surgery and neurology were predominant in men, and admissions for internal medicine and trauma were predominant in women. Patients aged 65–79 years were admitted to internal medicine, and those over 80 years were admitted to cardiology. Among patients who presented to the hospital’s emergency department and required admission, 51.5% were men aged ≥ 75 years, rising to 53.3% among those aged 65 to 74 years. The clinical areas were related to cardiology (27.67%) and pneumology (20.63%). **Conclusions**: Demands for ED care in those over 65 years of age are associated with sociodemographic and clinical characteristics, which can be used to better plan and manage resources and improve user satisfaction.

## 1. Introduction

Hospital emergency departments (EDs) are the apex of the emergency care pyramid, where patients treated at the other levels of the healthcare network converge with those who come on their own initiative [1]. They serve as a critical point of access to the healthcare system, ensuring immediate medical attention for acute conditions, chronic disease exacerbations, and injuries.

Over the last three decades, EDs have been under increasing pressure to provide care [2]. This growing demand is driven by multiple factors, including population aging, the increased prevalence of chronic diseases, and rising patient expectations. Several studies and scientific societies have attempted to characterize the profile of patients over 65 years of age who present to EDs, noting that this group more frequently presents with nonspecific symptoms (such as weakness, confusion, or falls), which sometimes mask serious underlying pathologies. This has generated debates about ED overcrowding, its causes, consequences, or implications, and possible solutions [3], in addition to a number of internal factors considered by the American College of Emergency Physicians [4] related to the functioning of the ED itself. There are also several general external parameters that influence ED utilization, such as pollution levels [5], influenza epidemics [6], atmospheric changes [7,8,9], lunar cycles [10], and sporting events [11].

The characteristics of the population served by each ED are among the essential factors to consider, and in particular, the population aged 65 years or older (older adults) deserves special attention, since they represent approximately 25% of the total ED visits [12], tend to have multiple chronic diseases, polypharmacy, and functional decline, which complicates both triage and clinical management. Furthermore, it has been observed that older adults are more likely to experience prolonged wait times, repeated visits, and adverse outcomes, including hospital readmissions and mortality, generate a higher percentage of diagnostic tests (50% more than other patients), have a 20% longer hospital stay, are more likely to be admitted (20.4% vs. 6.9%) [12] and, in addition, are classified as an appropriate emergency in a higher percentage than the population under 65 years of age (48.2% vs. 35.5%) [13]. Therefore, this growing population group must be considered when planning and seeking solutions to current urgent healthcare problems [14,15,16].

In 2022, 19.25% of the Spanish population was aged 65 years and over, and 6.1% were aged over 80 years [17], with an upward trend; this trend has a direct effect on ED attendance. Furthermore, the number of visits to the ED increases by five-year age groups, especially from the age of 65 years [18].

In high-income countries such as Canada, the United Kingdom, and Australia, a sustained and increasing trend in ED visits by people over 65 years of age has been observed, leading to the implementation of specific geriatric care models in EDs.

Compared to these countries, Spain has a different healthcare structure and less development of specific protocols for older adults in EDs, underscoring the need to adapt care strategies to their growing demand and clinical complexity. The current gaps in knowledge about emergency care for people over 65 years of age and the increasing demand for them not only affect hospital capacity but also underscore the importance of developing preventive strategies, strengthening primary care, and improving home care alternatives to reduce unnecessary emergency department visits.

For these reasons, it is important to identify predictive factors surrounding emergency care for patients aged 65 years and older to align resources with demand. The need to improve understanding of the specific characteristics of this population group and promote training and knowledge sharing among healthcare professionals who care for this group can improve clinical decision-making, reduce unnecessary admissions, and improve patient outcomes. The objective of this study was to identify the underlying characteristics of this care, establishing a profile of these patients that will allow us to analyze factors such as morbidity, clinical factors, healthcare specialties, outcomes, and administrative characteristics of demand.

## 2. Materials and Methods

### 2.1. Design and General Aspects

This was a cross-sectional descriptive study of the sociodemographic, clinical, and service utilization variables recorded in the office records of the Emergency Admission application of the hospital of Mérida for the selected age group.

It was conducted in the health area of Mérida, which has an ED attendance rate of 1.6 for people aged 65 and over, which is higher than that of Extremadura (the highest Autonomous Community in the country with 1.47) and far from the National Health System (NHS) average of 0.66. Furthermore, the area of the Mérida region has a rate of overage (>80/>65×100) of 17.7%, which is also higher than the NHS average of 17.5% for 2019 [18].

### 2.2. Population and Sample: Population Characteristics

The study included all ED visits at Mérida in 2019 (the last year before the Coronavirus Disease 2019 [COVID-19] pandemic) for patients aged 65 years and older (*N* = 5846). A total of 12.72% of the cases are undefined or illegible (their exclusion was not necessary, because an illegible or indeterminate diagnosis did not modify the treatment of sociodemographic, clinical, and administrative variables). This figure is lower than the 13.8% with ill-defined symptoms and signs reported in another study [13].

### 2.3. Data Collection

The data collected corresponded to visits in the ED of Mérida between 00:00 h on 1 January and 23:59 h on 31 December 2019 among users aged 65 years and over. The data included in the file are of administrative and clinical origin and are provided by the IT service from the Emergency Admission Register after authorization by Area Management.

### 2.4. Instruments and Data Processing System

Before starting the study itself and its respective statistical analysis, the database was sorted and cleaned in the variables with free text fields to achieve coherence and grouping of some concepts that appeared under different names or with spelling errors. The data harmonization process was performed using a manual and automated approach combined with Microsoft Excel and SPSS (version 25). The criteria used to group or recode variables such as the reason for discharge or diagnoses were based on the Minimum Basic Data Set (MBDS) at discharge.

### 2.5. Variables

The variables analyzed were grouped into three main categories: sociodemographic, clinical, and administrative. In terms of sociodemographic variables, the patient’s gender, the reference population of the health area of origin (rural or urban), and age, calculated from the date of birth, were recorded. Clinical variables included the recorded diagnosis and the medical specialty involved in the care. The administrative variables considered were the date and time of admission (to determine the shift (morning, afternoon, night), the day of the week (weekday or holiday), the month, and the season of the year), as well as the date and time of discharge, which allowed the length of stay in the emergency department to be calculated. The reason for discharge from the emergency department was also recorded (home, hospitalization, outpatient consultation, transfer, voluntary discharge, death, absconding, nonurgent, or undetermined), as well as the initial pathology recorded by the administrative service (accidental injury, common illness, animal bite, assault, self-harm, poisoning, and others). All identifying variables of the subjects (surnames and first names) were anonymized at all times and replaced by an identification number, which allowed the frequency of the phenomenon to be observed in aggregate form without compromising the confidentiality of the data.

### 2.6. Data Analysis

The data were statistically processed and analyzed via the Statistical Package for Social Sciences (SPSS, version 25.0; Armonk, NY, USA). Descriptive statistics were computed for all the dependent variables. Specifically, data on the number of hours spent in the ED are presented as the mean and standard deviation, whereas data on the total number of ED visits, reasons for discharge, and diagnostic specialties are presented as absolute frequencies (absolute values) and relative frequencies (percentages of the total). Descriptive data were calculated for the total sample and stratified by gender, season of the year, age group, day type (working day or holiday), shift, and population area (rural or urban). The normality of the data was tested via the Shapiro–Wilk test. The Mann–Whitney *U* test was then used to compare each of the dependent variables, considering the stratifications carried out. The alpha level was set at *p* < 0.05.

### 2.7. Ethical Aspects

It is not necessary to apply data protection principles to anonymous information, which cannot be linked to identifiable individuals. Regulation (EU) 2016/679 of the European Parliament and Council of 27 April 2016, which protects individuals regarding the processing of their personal data and the free movement of such data, does not affect the processing of the information in this study. Furthermore, the use of this information for statistical or research purposes does not require the approval of an accredited ethics committee. In any case, the study was conducted in accordance with the Helsinki Declaration and local institutional guidelines.

## 3. Results

There were 5846 visits to patients aged 65 years and over during the year. Differences were found between the number of ED hours and gender (*p* < 0.001), season (*p* = 0.024), age group (*p* < 0.001), type of care (*p* < 0.001), and population area (*p* = 0.003) (Table 1).

**Figure 1:**
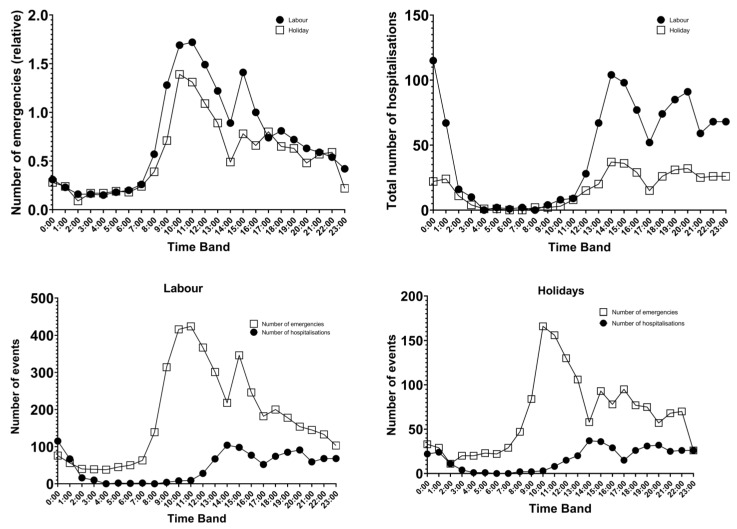
Number of emergencies and hospitalizations by type of day and time slot.

Emergencies by time slot between working hours and public holidays (parallel curves) follow the same pattern, except that relatively fewer emergencies are attended on public holidays than during working hours. By time slot, the lowest number of episodes occurs between 2:00 and 3:00, and the maximum occurs between 10:00 and 11:00 and at 16:00. Hospital admissions by time slot and between working days and public holidays followed the same pattern as ED visits did (Figure 1).

The most common reason for discharge from the emergency department was discharge to home at the end of treatment (63.5%) or transfer to the hospital (admission) (25.7%) (Table 2).

Differences of more than 1% are found in some clinical areas according to different variables: (a) gender: in men, they are found in surgery-digestive, pneumology, oncology and urology, and in women, in internal medicine, rheumatology and traumatology (Table 3A); (b) age group: between 65 and 79 years in internal medicine, ophthalmology, otorhinolaryngology (ENT), and traumatology, resulting in favor of those ≥80 years in cardiology, surgery-digestive, pneumology, neurology, and urology (Table 3A); (c) type of day: on working days ophthalmology and traumatology for holidays (Table 3B); (d) type of population: only in urban areas for internal medicine and ENT (Table 3B); (e) season of the year: resulting in differences in pneumology (higher in winter) and traumatology (higher in summer) (Table 3C); (f) shift: higher in the night shift in cardiology, internal medicine, and pneumology; higher in the afternoon shift in rheumatology and traumatology; and higher in the morning shift in ophthalmology (Table 3C).

Overall, the three most common diagnoses are contusion, infection, and heart failure. In terms of gender, these include heart failure, chronic obstructive pulmonary disease, and ischemic heart disease in men and contusion, fracture, and heart failure in women. By age group, different diagnoses appear for those aged 65–79 years (contusion, infection, and chest pain) and those >80 years (heart failure, infection, and contusion). By the season of the year, in spring and summer, contusion, heart failure, and fracture occur; in autumn, contusion, fracture, and infection occur; and in winter, infection, contusion, and heart failure occur (Table 4).

Patients presenting to the hospital emergency departments requiring admission had the following profile: 53.8% ≥75 years, 51.5% men (53.3% aged 65–74 years), and the associated clinical areas were cardiology (27.67%), and pulmonology (20.63%) (Table 5).

## 4. Discussion

The results obtained in this study indicate the significant use of EDs by the population ≥ 65 years of age and allow us to identify predictive factors surrounding the demand for urgent care, enabling us to match resources to demand.

EDs overuse is a universal problem that requires rethinking the healthcare system and EDs. The causes of overcrowding are many and include both external and internal aspects, the most important being those inherent in the dynamics of the hospital [19]. In our case, we have focused on the objective manifestations that have an impact on the service causing this saturation, which, from our point of view, should not be associated in many aspects with the intrinsic determinants of the ED without considering other characteristics of the specificity of the users (clinical or otherwise). Thus, the results demonstrate the influence of nonclinical factors, such as sociodemographic and other clinical characteristics of prevalent morbidity, on ED use [20,21,22].

Despite being a health area with one of the highest ED attendance rates in the country for patients aged 65 years and over [18] and with an overage rate also higher than the Spanish National Health System average [18], our data show lower ED use and fewer episodes than those of other studies [19,23] because they focus only on hospital emergencies. The characteristics surrounding ED attendance were patients approximately 75 years of age, mainly women, attending mainly on working days around mid-morning or early afternoon, mainly in winter, and mostly from urban areas. However, in our study, we found a significant difference in ED visits according to urban (>50,000 inhabitants) or rural (*p* = 0.003) residence, in line with Prado-Galbarro et al. [23]; all of this was because many records are due to multiple visits by the same users. Thus, according to the classifications made by Castillo et al. [24] or Vinton et al. [25], in our study, we found 0.24% hyperfrequent users, 8.45% normal users, and 91.31% infrequent users.

The population aged ≥ 80 years, despite having more pathologies and greater frailty, is associated with lower relative use (26% of those aged ≥ 80 years) than those aged 65–79 years (51.7% of those in this group), and women use these services less than men do, although in absolute terms they constitute the majority in terms of the number of visits [26], probably due to their different life expectancies.

Although some patients use EDs for minor (nonurgent) conditions, we can observe that frequent users are often patients with multiple chronic diseases associated with high admission and mortality rates [27,28], we propose including case management for high-frequency users and improving access to primary care. Nevertheless, our percentages of high- and normal-frequency users are low for the age group studied, and we have a high number of low-frequency users [24,25]; women aged approximately 73 years and those living in urban areas are more likely to be high-frequency ED users. The higher use by urban women could reflect a smaller support network or a higher comorbidity burden. The mean number of ED visits was greater in the young population than in the older population [23].

Both the number of emergencies and the number of admissions show similar behavior, with no differences with respect to the type of day (working day or holiday) with a few-hour delay between care and admission. In terms of time slots, there are increases in cases at midday, approximately 3 pm and in the early afternoon, which is identical to previous studies that considered all age groups [13]; thus, it can be deduced that the times of day when it is decided to visit the emergency department, except for those of a vital nature, are shaped by social customs and habits.

The time spent in the ED differs significantly only during the night shift compared with any other shift (morning–afternoon) because of the patients’ resting time and the reduced demand on the ED and central services (laboratory, radiology, etc.), but not with regard to the day of the week or whether it is a working day or a public holiday, and the difference in the average time spent in the ED until care is resolved between men (7.2 ± 6.95 h) and women (6.55 ± 6.94 h) is significant.

The four most common diagnostic groups, accounting for 31.45% of emergency visits, were: (1) injuries (≈10%), primarily due to contusions, fractures, wounds, trauma, and sprains; (2) cardiovascular diseases (≈8.75%), including heart failure, stroke, arterial hypertension, and atrial fibrillation; (3) respiratory diseases (≈7.2%), particularly during winter, such as chronic obstructive pulmonary disease, respiratory infections, pneumonia, respiratory failure, and bronchitis; and (4) ill-defined symptoms and signs (≈5.5%), often related to abdominal and chest pain. These findings are consistent with those of Pozuelo García et al. [13], although their study included a population of all ages. Similar patterns have been reported in other healthcare systems experiencing accelerated population aging. Notably, a substantial proportion of cases were classified as indeterminate (7.37%) or illegible (5.34%).

The patients who had to be admitted were mainly patients aged ≥ 75 years, men, and admitted to the clinical areas of cardiology, pneumology, gastrointestinal surgery, and neurology, in that order, with slightly different results from those obtained in the study by Pozuelo García et al. [13]; this was undoubtedly due to different nuances in the diagnostic grouping, which, in our case, proved to be more exhaustive and to extend, in that study, to all types of emergencies.

Detailed knowledge of clinical (morbidity and its characteristics), demographic (age, gender, marital status, etc.), social (education, occupation, religion, ethnicity, etc.), family (number of members, previous experience of the disease, etc.), individual (severity, self-management, etc.), and community (price and access to health services, autonomous community, etc.) perceptions surrounding ED care will allow us to generate models with certain ranges of ED demand forecasts that contribute to improving health planning in this essential service [29,30,31].

The planning of material and human resources must consider the factors analyzed in this study (nonclinical and clinical), since the phenomenon of population aging together with the increase in chronic morbidity and dependency of these patients is a progressive and unstoppable phenomenon in developed countries [32]. Although some authors have highlighted the need to create an ED specialty [33,34], we believe, in agreement with Puig-Campany, that it is more likely that EDs will be adapted to the care of elderly patients [2] and even other groups, such as pediatric, adolescent, and adult patients.

The appropriateness of emergency room use remains a complex concept, influenced by clinical criteria, patient perception, and triage systems. Specific training for staff in geriatric care could improve the response to the needs of this group, and an analysis of the relationship with healthcare personnel can be a decisive component of the care experience [35].

This study has several limitations. First, as it is a cross-sectional study, causality cannot be established. However, the results are consistent with those of previous studies. Second, some variables, such as diagnoses, and others based on diagnoses, such as clinical area and specialty, are subject to potential bias, as 12.72% of diagnoses are undefined or illegible cases, and we have not been able to determine whether these cases occur proportionally in all clinical areas and specialties. Furthermore, although they were not the main focus of this study, some relevant quality indicators in the care of older adults in emergency departments were considered transversally, such as the return visit rate (hyperfrequency) and in-hospital mortality in the ED, whose more detailed exploration could be the subject of future specific analyses. Finally, the inability to use a standardized coding system for diagnoses (e.g., International Classification of Diseases, 10th Revision or Diagnosis Related Groups) has limited comparisons with other studies, although these were not used either. Future research could incorporate prospective designs, standardized diagnostic coding, or qualitative methods focused on the patient experience.

However, the main strengths are that the reported medical diagnosis is derived from the clinical history of all patients seen in the ED over one year, and the results are comparable to those of similar populations and correspond to the last prepandemic year of the COVID-19 pandemic, which opens the door for use as a reference for postpandemic comparative studies. This work opens the door to the development of predictive demand models in the ED that will allow us to anticipate the demand at any given time.

## 5. Conclusions

These findings reinforce the urgent need for age-adapted emergency services and data-driven resource planning in aging societies. Specific utilization profiles were identified, with more patients aged approximately 75 years and a predominance of women, although men were more frequent attenders. The peak use was observed during daytime hours, and there were seasonal variations, particularly in winter. The main diagnoses included injuries, cardiovascular disease, respiratory problems, and unspecified symptoms, reflecting the morbidity of this population. In addition, predictive factors were identified that may help in the development of demand models. These findings are particularly relevant in view of the progressive aging of the population and increasing chronic morbidity in developed countries, provide a valuable benchmark for future comparisons and suggest the need to adapt emergency services to the specific characteristics of the elderly population, thus optimizing the planning and allocation of healthcare resources.

## Figures and Tables

**Table 1:** Descriptive statistics and number of hours of stay in the ED for the whole sample and stratified by variable.

Ranking	Categories	*N*		Number of Hours in the Emergency Room		*p*-Value
		*n*	% of Total	*M* (Min/Max)	SD	
General	Total	5846	100	6.85 (0.02/97.15)	6.95	-
Gender	Man	2650	45.33	7.20 (0.02/95.30)	6.95	**<0.001**
	Woman	3196	54.67	6.55 (0.02/97.15)	6.94	
Season year	Spring	1482	25.35	6.55 (0.02/75.20)	6.06	**0.024**
	Summer	1496	25.59	6.98 (0.02/95.30)	6.92	
	Autumn	1301	22.25	6.39 (0.02/79.38)	6.35	
	Winter	1567	26.80	7.38 (0.03/97.15)	8.11	
Age group	65–79 years	4339	74.22	6.58 (0.02/97.15)	6.86	**<0.001**
	≥80 years	1507	25.78	7.61 (0.02/95.30)	7.16	
Type of day	Labor (260)	4273	73.09	6.82 (0.02/95.30)	6.71	0.098
	Holiday (105)	1573	26.91	6.91 (0.02/97.15)	7.57	
Attention shift	Morning (8:00–15:00)	2926	50.05	6.56 (0.02/97.15)	7.53	**<0.001**
	Afternoon (15:00–22:00)	1994	34.11	6.40 (0.02/83.02)	6.14	
	Night (22:00–8:00)	926	15.84	8.71 (0.02/40.25)	6.36	
Population area	Rural (2874)	1307	22.36	7.43 (0.02/97.15)	8.18	**0.003**
	Urban (9846)	4539	77.64	6.68 (0.02/95.30)	6.55	

Bolded values denote statistical significance (*p* < 0.05). Min, minimum; Max, maximum; SD, standard deviation.

**Table 2:** Discharge destination, broken down by age group.

	65–79		>80		Both Groups	
	*n*	%	*n*	%	*n*	%
Home discharge (end of care)	2888	65.10	827	58.8	3715	63.50
Transfer to hospitalization (admissions) (a)	1048	23.60	453	32.2	1501	25.70
Referral to outpatient	406	9.20	89	6.30	495	8.50
Transfer to another hospital (b)	35	0.80	7	0.50	42	0.70
Exitus	20	0.36	15	1.10	35	0.60
Hospitalization at home (c)	9	0.20	4	0.30	13	0.20
Patient elopement (d)	2	0.04	-	-	2	0.04
Undetermined/other	31	0.70	12	0.80	43	0.76
Total	4439	100.00	1407	100.00	5846	100.00

(a) Transfer from emergency unit to a medical specialty of the center. (b) Hospital of higher technical complexity. (c) Patient resides at home and the healthcare team travels to the patient’s home to provide healthcare. (d) Patient leaves the center without any medical indication or order and without the knowledge of the staff.

**Table 3:** (**A**) Percentage of ED visits by clinical specialty, gender, and age group. (**B**) Percentage of ED visits by clinical specialty, type of day, and type of population. (**C**) Percentage of ED visits by clinical specialty, season, and shift of care.

Panel A																				
	TOTAL (*N* = 5846)					Gender									Age Group					
						Men (*n* = 2650)				Women (*n* = 3196)					65–79 (*n* = 4339)			≥80 (*n* = 1507)		
	*n*			%		*n*			%	*n*		%		*n*		%		*n*		%
Traumatology	1007			17.23		300			11.32	707		22.12		786		18.11		221		14.66
Indeterminate (a)	944			16.15		431			16.26	513		16.05		717		16.52		227		15.06
Cardiology	624			10.67		284			10.72	340		10.64		419		9.66		205		13.60
Surgery/digestive	589			10.08		286			10.79	303		9.48		407		9.38		182		12.08
Pneumology	588			10.06		363			13.70	225		7.04		420		9.68		168		11.15
Internal medicine	328			5.61		132			4.98	196		6.13		271		6.25		57		3.78
Urology	322			5.51		211			7.96	111		3.47		226		5.21		96		6.37
Neurology	284			4.86		140			5.28	144		4.51		197		4.54		87		5.77
Otorhinolaryngology	214			3.66		98			3.70	116		3.63		175		4.03		39		2.59
Ophthalmology	195			3.34		88			3.32	107		3.35		162		3.73		33		2.19
Rheumatology	141			2.41		48			1.81	93		2.91		105		2.42		36		2.39
Oncology	126			2.16		83			3.13	43		1.35		95		2.19		31		2.06
Vascular	108			1.85		47			1.77	61		1.91		79		1.82		29		1.92
Psychiatry	91			1.56		47			1.77	44		1.38		78		1.80		13		0.86
Endocrinology	74			1.27		25			0.94	49		1.53		51		1.18		23		1.53
Neurosurgery	52			0.89		19			0.72	33		1.03		40		0.92		12		0.80
Gynecology	44			0.75		-			-	44		1.38		37		0.85		7		0.46
Hematology	38			0.65		9			0.34	29		0.91		17		0.39		21		1.39
Nephrology	37			0.63		21			0.79	16		0.50		23		0.53		14		0.93
Dermatology	28			0.48		14			0.53	14		0.44		22		0.51		6		0.40
Dentistry	6			0.10		2			0.08	4		0.13		6		0.14		-		-
Plastic surgery	5			0.09		1			0.04	4		0.13		5		0.12		-		-
Maxillofacial	1			0.02		1			0.04	-		-		1		0.02		-		-
**Panel B**																				
	**TOTAL** **(*N* = 5846)**						**Day Type**								**Population**					
							**Labor** **(** ***n* = 4273)**				**Holiday** **(** ***n* = 1573)**				**Rural** **(** ***n* = 1307)**			**Urban** **(** ***n* = 4539)**		
	***n***				**%**		**%**				**%**				**%**			**%**		
Traumatology	1007				17.23		16.78				18.44				17.52			17.14		
Indeterminate (a)	944				16.15		16.15				16.15				15.76			16.26		
Cardiology	624				10.67		10.48				11.19				11.25			10.51		
Surgery/digestive	589				10.08		10.30				9.47				10.86			9.85		
Pneumology	588				10.06		10.27				9.47				10.02			10.07		
Internal medicine	328				5.61		5.43				6.10				4.28			5.99		
Urology	322				5.51		5.66				5.09				5.81			5.42		
Nephrology	284				4.86		4.68				5.34				4.74			4.89		
Otorhinolaryngology	214				3.66		3.72				3.50				2.83			3.90		
Ophthalmology	195				3.34		3.77				2.16				3.98			3.15		
Rheumatology	141				2.41		2.39				2.48				2.30			2.45		
Oncology	126				2.16		2.43				1.40				2.07			2.18		
Vascular	108				1.85		1.85				1.84				1.91			1.83		
Psychiatry	91				1.56		1.31				2.23				1.30			1.63		
Endocrinology	74				1.27		1.12				1.65				1.38			1.23		
Neurosurgery	52				0.89		0.94				0.76				1.15			0.82		
Gynecology	44				0.75		0.80				0.64				0.84			0.73		
Hematology	38				0.65		0.66				0.64				0.92			0.57		
Nephrology	37				0.63		0.54				0.89				0.61			0.64		
Dermatology	28				0.48		0.59				0.19				0.31			0.53		
Dentistry	6				0.10		0.07				0.19				0.08			0.11		
Plastic surgery	5				0.09		0.05				0.19				0.08			0.09		
Maxillofacial	1				0.02		0.02				-				-			0.02		
**Panel C**																				
		**TOTAL** **(*N* = 5846)**			**Season**										**Shift of Care**					
					**Spring** **(*n* = 1482)**			**Summer** **(*n* = 1496)**		**Autumn (*n* = 1301)**			**Winter** **(*n* = 1567)**		**Morning** **(*n* = 2926)**		**Afternoon** **(*n* = 1994)**		**Night** **(*n* = 926)**	
		***n***	**%**		**%**			**%**		**%**			**%**		**%**		**%**		**%**	
Traumatology		1007	17.23		18.56			18.65		17.14			14.68		17.29		19.86		11.34	
Indeterminate (a)		944	16.15		13.56			21.26		12.91			16.40		16.20		15.65		17.06	
Cardiology		624	10.67		11.40			9.89		11.38			10.15		9.98		9.13		16.20	
Surgery/digestive		589	10.08		10.19			10.49		9.92			9.70		9.54		10.43		11.02	
Pneumology		588	10.06		9.85			5.08		9.68			15.32		9.81		9.18		12.74	
Internal medicine		328	5.61		5.67			4.75		6.53			5.62		5.40		5.22		7.13	
Urology		322	5.51		5.80			5.68		5.30			5.23		4.85		6.12		6.26	
Nephrology		284	4.86		4.86			4.48		5.38			4.79		4.78		4.91		4.97	
Otorhinolaryngology		214	3.66		3.91			3.28		3.77			3.70		4.14		2.91		3.78	
Ophthalmology		195	3.34		3.31			3.34		3.23			3.45		4.89		2.41		0.43	
Rheumatology		141	2.41		2.09			2.81		3.00			1.85		2.63		2.86		0.76	
Oncology		126	2.16		2.83			1.87		2.46			1.53		2.46		1.91		1.73	
Vascular		108	1.85		1.69			2.01		2.15			1.60		1.40		2.61		1.62	
Psychiatry		91	1.56		1.21			1.80		1.69			1.53		1.50		1.65		1.51	
Endocrinology		74	1.27		1.42			1.27		1.46			0.96		1.09		1.55		1.19	
Neurosurgery		52	0.89		0.61			0.67		1.15			1.15		0.79		1.15		0.65	
Gynecology		44	0.75		1.01			0.60		1.08			0.38		0.92		0.80		0.11	
Hematology		38	0.65		0.67			0.27		0.92			0.77		0.82		0.60		0.22	
Nephrology		37	0.63		0.74			0.87		0.23			0.64		0.55		0.65		0.86	
Dermatology		28	0.48		0.40			0.74		0.38			0.38		0.68		0.25		0.32	
Dentistry		6	0.10		0.13			0.07		0.15			0.06		0.10		0.15		-	
Plastic surgery		5	0.09		0.07			0.13		-			0.13		0.14		-		0.11	
Maxillofacial		1	0.02		-			-		0.08			-		0.03		-		-	

(a) The specialty is not stated or cannot be deduced from the injury, sign, or symptom treated.

**Table 4:** Percentages of diagnoses in ED care by gender, age group, and season.

	Total (%)	Gender (%)		Age (%)		Season (%)			
		Men	Women	65–79	≥80	Spring	Summer	Autumn	Winter
	*N* = 5846	(*n* = 2650)	(*n* = 3196)	(*n* = 4339)	(*n* = 1507)	(*n* = 1482)	(*n* = 1496)	(*n* = 1301)	(*n* = 1567)
Indeterminate ^a^	7.37	7.47	7.29	7.88	5.91	8.43	8.89	6.46	6.83
Illegible	5.35	5.55	5.19	5.28	5.57	4.12	7.69	2.69	5.36
Contusion	4.43	2.60	5.94	4.75	3.52	4.93	4.08	4.38	4.34
Infection	3.66	4.23	3.19	3.34	4.58	3.10	1.74	3.38	6.25
Heart failure	3.51	3.25	3.72	2.72	5.77	4.05	3.28	2.92	3.7
Fracture	3.23	2.08	4.19	2.60	-	3.58	3.01	3.38	3.00
Chest pain	2.94	2.72	3.13	3.32	1.86	2.43	2.87	3.00	3.45
Ischemic heart disease	2.89	3.28	2.57	3.02	2.52	2.97	2.94	3.15	2.55
Abdominal pain	2.46	2.57	2.38	2.70	1.79	2.56	2.74	2.69	1.91
Nonurgent ^b^	2.41	1.96	2.78	2.47	2.26	2.33	2.47	2.15	2.74
Cerebrovascular accident	2.16	1.78	2.60	1.91	2.85	1.82	1.87	2.31	2.62
Neoplasia	2.12	3.09	1.31	2.17	1.99	2.77	1.87	2.46	1.47
COPD	1.92	3.96	<1.00	2.17	1.19	<1.00	1.00	1.92	3.06
Vertigo	1.64	1.28	<1.00	1.84	1.06	1.82	1.40	1.61	1.72
Pneumonia	1.59	2.08	1.19	1.36	2.26	1.96	1.14	1.08	2.11
High blood pressure	1.56	1.32	1.75	1.77	<1.00	1.28	1.00	2.38	1.66
Atrial fibrillation	1.52	1.36	1.66	1.59	1.33	1.69	1.00	1.84	1.60
UTI	1.45	1.43	1.47	<1.00	2.19	1.55	1.54	1.54	1.21
Syncope	1.30	1.25	1.35	<1.00	2.19	1.69	1.40	1.38	<1.00
Arthrosis	1.28	<1.00	1.66	1.36	1.06	1.01	1.74	1.46	<1.00
Hemorrhage	1.11	1.43	<1.00	1.08	1.19	1.28	<1.00	<1.00	1.28
Nephritic colic	1.06	1.06	1.06	1.29	<1.00	1.08	1.27	<1.00	1.08
Arthritis	1.04	<1.00	1.19	1.15	<1.00	<1.00	1.20	1.15	<1.00
Biliary colic	1.04	<1.00	1.16	1.18	<1.00	1.08	1.20	1.15	<1.00
Anxiety	1.03	1.09	<1.00	1.24	<1.00	<1.00	1.40	1.23	<1.00
Cephalalgia	<1.00	<1.00	1.19	1.18	<1.00	1.21	1.14	<1.00	<1.00
Hematuria	<1.00	<1.00	<1.00	<1.00	1.19	1.21	1.00	<1.00	<1.00
Wound	<1.00	<1.00	1.03	1.08	<1.00	1.08	<1.00	1.15	<1.00
Lumbalgia	<1.00	<1.00	1.28	1.08	<1.00	1.08	1.20	<1.00	<1.00
Sprain	<1.00	<1.00	1.22	1.06	<1.00	<1.00	1.47	<1.00	<1.00
Traumatism	<1.00	<1.00	<1.00	<1.00	<1.00	<1.00	1.00	<1.00	<1.00
Respiratory distress	<1.00	1.06	<1.00	<1.00	1.46	<1.00	<1.00	1.38	1.02
Bronchitis	<1.00	<1.00	<1.00	<1.00	<1.00	<1.00	<1.00	<1.00	1.28
Intestinal obstruction	<1.00	<1.00	<1.00	<1.00	1.00	<1.00	<1.00	<1.00	1.02
Anemia	<1.00	<1.00	<1.00	<1.00	1.26	<1.00	<1.00	<1.00	<1.00
Constipation	<1.00	<1.00	<1.00	<1.00	1.13	<1.00	<1.00	<1.00	<1.00
TIA	<1.00	<1.00	<1.00	<1.00	1.06	<1.00	<1.00	<1.00	<1.00
Urinary retention	<1.00	1.40	<1.00	<1.00	<1.00	<1.00	<1.00	<1.00	<1.00

^a^ Physicians do not specify a diagnosis or specify signs and/or symptoms instead of a diagnosis; ^b^ nonurgent according to medical records. COPD, chronic obstructive pulmonary disease; TIA, transient ischemic attack; UTI, urinary tract infection. Note: Shaded rows correspond to non-diagnostic or non-pathological categories included for organizational clarity.

**Table 5:** Percentage of admissions by clinical area, gender, age group, and age group by gender.

		Gender		Age Group		65–74		≥75	
	Total (*N* = 1501)	Man (*n* = 773)	Woman (*n* = 728)	65–74 (*n* = 694)	≥75 (*n* = 807)	Man (*n* = 412)	Woman (*n* = 282)	Man (*n* = 338)	Woman (*n* = 446)
	%	%	%	%	%	%	%	%	%
Cardiology	27.91	26.39	29.53	27.09	28.62	27.67	26.24	19.82	31.61
Surgery/digestive	12.13	11.64	12.64	11.96	12.27	11.41	12.77	12.72	12.56
Dermatology	0.20	-	0.41	0.14	0.25	-	0.35	-	0.45
Endocrinology	1.00	1.03	0.96	1.01	0.99	0.73	1.42	1.48	0.67
Gynecology	0.87	-	1.79	1.30	0.50	-	3.19	-	0.90
Hematology	1.20	0.65	1.79	1.15	1.24	0.73	1.77	0.59	1.79
Indeterminate ^a^	5.46	6.47	4.40	6.05	4.96	6.80	4.96	6.51	4.04
Internal medicine	3.20	2.59	3.85	3.31	3.10	2.43	4.61	2.96	3.36
Nephrology	1.40	1.81	0.96	1.01	1.73	0.97	1.06	2.96	0.90
Pulmonology	17.26	20.31	14.01	17.15	17.35	20.63	12.06	21.30	15.25
Neurosurgery	1.00	0.78	1.24	0.86	1.12	0.73	1.06	0.89	1.35
Neurology	10.46	10.35	10.58	10.81	10.16	10.19	11.70	11.24	9.87
Dentistry	0.07	-	0.14	0.14	-	-	0.35	-	-
Ophthalmology	0.93	0.78	1.10	1.01	0.87	1.21	0.71	0.30	1.35
Oncology	5.26	7.12	3.30	6.63	4.09	7.52	5.32	7.10	2.02
Otorhinolaryngology	0.87	0.91	0.82	1.30	0.50	1.21	1.42	0.59	0.45
Psychiatry	0.27	0.13	0.41	0.14	0.37	-	0.35	0.30	0.45
Rheumatology	0.47	0.65	0.27	0.43	0.50	0.73	-	0.59	0.45
Traumatology	6.00	3.62	8.52	4.18	7.56	2.43	6.74	5.33	9.64
Urology	2.07	2.59	7.96	2.74	1.49	3.16	2.13	2.07	1.12
Vascular	2.00	2.20	1.77	1.59	2.35	1.46	1.77	3.25	1.79

^a^ The specialty is not stated or cannot be deduced from the injury, sign, or symptom treated.

## Data Availability

Datasets are available through the corresponding author upon reasonable request.
